# Testing the predictions of the interpersonal-psychological theory of suicide in a sample of female cancer patients

**DOI:** 10.1007/s00520-025-10224-2

**Published:** 2025-12-02

**Authors:** Jan Schomberg, Tobias Teismann, Alexander L. Gerlach, Jan C. Cwik

**Affiliations:** 1 Department of Clinical Psychology and Psychotherapy, University of Cologne, Cologne, North Rhine-Westphalia Germany; 2 Department of Psychology, Mental Health Research and Treatment Center, Ruhr-University of Bochum, Bochum, North Rhine-Westphalia Germany; 3 Faculty of Health Care, Hochschule Niederrhein, Krefeld, North Rhine-Westphalia Germany

**Keywords:** Suicide, Interpersonal-psychological theory, Oncology, Perceived burdensomeness, Cancer, Thwarted belongingness

## Abstract

**Objectives:**

Individuals suffering from cancer have a heightened risk of suicide; thus, understanding suicide-related thoughts and behaviors is key to identifying vulnerable patients. The interpersonal theory of suicide (IPTS) provides a comprehensive framework for examining suicide risk factors and has been validated across a range of samples. This study aimed to evaluate the applicability of IPTS in a group of female cancer patients.

**Methods:**

This study was registered with the German Clinical Trials Register (DRKS00020477; registered on June 30, 2020). In this study, we tested three major hypotheses formulated by the IPTS, using a sample of 199 female cancer patients.

**Results:**

As predicted by the IPTS, heightened levels of thwarted belongingness (TB) and perceived burdensomeness (PB) were indicative of elevated levels of passive suicidal ideation (SI). Furthermore, the interaction of TB, PB, and hopelessness was predictive of active SI. However, contrary to theoretical assumptions, the findings indicate that an increased fear of death was associated with higher suicidal intent.

**Limitations:**

The cross-sectional design curtails the affirmation of hypotheses pertaining to causality. Rather than considering a measure of hopelessness specifically related to PB and TB, a general measure was employed. Additionally, the recruitment phase of the study coincided with the onset of the global SARS-CoV-2 virus pandemic, which might have impacted the results.

**Conclusions:**

Even though the three hypotheses were only partially verified, the IPTS provides a beneficial framework for health professionals who are caring for patients with cancer. Longitudinal studies are encouraged to further validate the theory and bolster its application in understanding suicidal behavior.

**Supplementary Information:**

The online version contains supplementary material available at 10.1007/s00520-025-10224-2.

## Introduction

Cancer diagnosis and treatment are associated with profound psychological distress, including elevated rates of suicidal ideation (SI) and behaviors, with prevalence estimates for SI ranging from 0.7 to 46.3% across studies [41]).

The risk of suicide among cancer patients is nearly twice that (28.6 per 100,000 person-years [51];) of the general US population (16.7 per 100,000 person-years [83],). The experience of cancer is inherently distressing [63], as patients confront not only the life-threatening nature of the disease but also the emotional and physical toll of treatment, including significant fears related to treatment side effects, cancer-associated pain, and physical deterioration [8, 62]. This heightened vulnerability to suicidality is influenced by multiple risk factors. General risk factors include mental disorders—notably depression, which is prevalent in cancer patients [34, 45, 66]—as well as previous suicide attempts, history of psychiatric hospitalization, prior suicidal ideation, low socioeconomic status [26], life stressors [43], and physical health conditions such as cancer [35]. Beyond these general factors, cancer-specific stressors play a pivotal role in elevating overall suicide risk. These include factors such as poor cancer prognosis, advanced disease stage, time since diagnosis, and specific chemotherapy agents [63] or lack of social support [44, 56].

Furthermore, tumor location is significantly associated with suicide risk in cancer patients; the highest risks are observed for cancers of the lung, head and neck, testes, bladder, and Hodgkin’s lymphoma (standardized mortality ratio > 5–10) [83]. However, it should be highlighted that different studies have identified varying tumor sites with the highest suicide risk (see [63], for a review).

Gender-specific factors also need to be discussed when considering the risk of suicide among cancer patients. Research indicates that female patients tend to experience more significant psychological distress and greater variability in social functioning (either better or worse) than males [84]. Additionally, a larger proportion of women engage in suicidal behavior, which is ultimately non-lethal compared to men [50]. Several studies indicate that women are more susceptible to risk factors linked to SI, including major depressive disorder, which they experience at twice the rate of men and a stronger relational focus, wherein women prioritize close relationships more than men and thus experience greater emotional distress when these connections fail [76]. Overall, the current clinical evaluation of SI and behavior, with the goal of a sensitive suicide risk prognosis, is insufficient [26]. Although psycho-oncology offers distress screening tools, there still exists a significant lack of specific resources for assessing suicide risk in cancer patients [83]. Despite the need for such resources, there remains a notable lack of studies examining the psychological risk and protective factors for suicide in cancer patients, as well as their complex interplay [20].

Given the scarcity of resources for identifying at-risk patients, the significant diversity of suicide risk factors among cancer patients, and the imperative to consider gender-specific factors, a comprehensive theoretical and empirical framework for evaluating suicide risk in this demographic is crucial. The interpersonal-psychological theory of suicide (IPTS) [38, 76] and its associated measures might offer such a framework. The IPTS appears particularly relevant in this field, as it considers socially pertinent factors such as existential needs [52], pain [37], and loneliness [17], all of which are vitally important for cancer patients. Complementing the IPTS, the integrated motivational-volitional model [58] further elaborates on the psychological processes underlying SI, with a focus on the roles of defeat, humiliation, and entrapment. While the integrated motivational-volitional model broadens our understanding of the transition from ideation to action through volitional factors (e.g., access to means, impulsivity), the IPTS remains remarkably relevant for cancer patients due to its emphasis on interpersonal dynamics, as cancer is a burden not only for those affected but also for family members and close personal contacts [27, 28]. Consequently, the IPTS serves as a useful theoretical model for exploring how interpersonal risk factors influence SI and behavior [76].

**Fig. 1:**
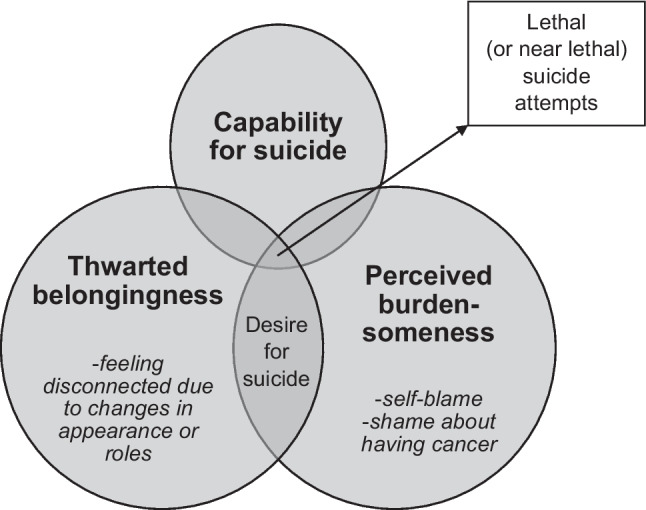
Factors of the IPTS and their relations. Indicators are specific examples for patients with cancer (following [76],adapted by the authors)

The IPTS proposes proximal risk factors for suicide, defined as “mental states and behavioral capacities evident to varying degrees in individuals at non-zero risk for lethal suicidal behavior” [76], p. 588). The IPTS suggests that these causal and interactive factors are necessary for the emergence of suicidal desire and capability [76]. The three main components of the IPTS are thwarted belongingness (TB), perceived burdensomeness (PB), and capability for suicide (Fig. 1). TB encompasses the dimensions of *loneliness* and the *absence of reciprocal care*. It is based on the theory asserting the human need to belong to others [7]. When this requirement goes unfulfilled, it can lead to the perception of being unvalued by one’s social group or family, which can potentially have negative health repercussions [9]. These effects may manifest as a desire to die (commonly known as passive SI), non-lethal suicidal behavior, and suicide [21]. The dimensions of TB include self-reported loneliness, possessing few or no friends, living alone, family conflict, and social withdrawal [76]. However, when the need to belong is met, it can deter suicidal actions, despite the presence of PB and capability for suicide [38].

PB refers to the potentially skewed perception that one’s existence is a burden to others and that one’s death might benefit society, friends, or family [38]. It comprises two dimensions: *liability* and *self-hate*. These dimensions of IPTS encompass distress resulting from physical illnesses as well as low self-esteem. Van Orden et al. [76] propose that PB is the common factor among family conflict, unemployment, and physical illness,all are associated with a heightened risk for suicide. Furthermore, PB might represent a maladaptive mental process stemming from evolutionarily adaptive behavior—that is, self-sacrifice for the protection of the species [39].

TB and PB alone are sufficient for passive SI (“I wish I were dead.”). Additionally, an active SI (“I want to kill myself.”) emerges when both components and the sense of hopelessness regarding changing those components are present [76]. Therefore, it is believed that heightened hopelessness concerning these two components significantly augments the risk of suicidal behavior.

Capability for suicide is the element distinguishing the desire to die from carrying out suicidal behavior [38]. To carry out suicidal behavior, a transformation from active SI to suicidal intent is hypothesized to be necessary. Suicidal intent can be defined as the degree of suicidal desire that is most likely to manifest into suicidal behavior (“I will kill myself”). Repeated exposure to painful and provocative experiences, such as physical pain, childhood maltreatment, combat exposure, or previous suicide attempts, is hypothesized to result in *a low fear of death* and *high physical pain tolerance*—both of which are relevant to the capability for suicide [76]. The underlying mechanisms are believed to be habituation and opponent processes, as suggested by opponent process theory [69], which both lead to reduced fear of self-harm [76]. The term *capability for suicide* evolved from the previously used term *acquired capability for suicide* to encompass both learned and genetic components of suicidal behavior due to the potential genetic element of suicide [13, 67].

In light of Van Orden et al.’s [76] request for validation of the theory, the IPTS has been empirically tested among a range of social groups such as firefighters [12], prison inmates [47], refugees [19], adolescents [71], older adults [75], sexual minorities [40], patients attending a psychiatry emergency department [5], and veterans/military personnel [3, 53]. However, although the WHO [81] predicts a significant rise in cancer prevalence by 2040, there is a lack of research on the relationship between IPTS and cancer [65]. Only individual aspects related to the IPTS, such as inadequate social support [56] or the general feeling of being a burden [1, 10, 79], have been assessed in populations of individuals with cancer. Villavicencio-Chávez et al. [77] suggested an association between psychological and physical impairment and the desire to hasten death in a sample of individuals with advanced cancer. Previous systematic reviews in this field have focused on the broad topic of suicide, researching incidence rates for suicide in patients with cancer, risk factors, or potential screening tools [4, 63, 70]. Additionally, SI in cancer patients has been analyzed [41].

To further scientific research, Van Orden et al. ([76], p. 581) proposed four falsifiable hypotheses, serving as a summary of the IPTS: “1. Thwarted belongingness and perceived burdensomeness are proximal and sufficient causes of passive suicidal ideation; 2. The simultaneous presence of thwarted belongingness and perceived burdensomeness, when perceived as stable and unchanging (i.e., hopelessness regarding these states), is a proximal and sufficient cause of active suicidal desire; 3. The simultaneous presence of suicidal desire and lowered fear of death serve as the condition under which suicidal desire will transform into suicidal intent; 4. The outcome of serious suicidal behavior (i.e., lethal or near-lethal suicide attempts) is most likely to occur in the context of thwarted belongingness, PB (and hopelessness regarding both), reduced fear of suicide, and elevated physical pain tolerance.”

The current study aims to test the initial three hypotheses of the IPTS within a cohort of female cancer patients. Investigation of the fourth hypothesis, which concerns the occurrence of lethal or near-lethal suicide attempts, falls outside the scope of this study due to the low occurrence rate of such events [55, 76]. The intention is to contribute to addressing the gap in IPTS literature about female cancer patients.

## Methods

### Participants and procedures

This research is a component of a larger longitudinal study that investigates the prevalence and associated characteristics of SI and suicidal behavior among German-speaking cancer patients who have completed a web-based survey. In Table 1, we summarize the sociodemographic characteristics as well as details regarding cancer types and treatment characteristics of the study sample. The study included *n* = 199 female cancer patients, with ages ranging from 18 to 77 years (*M* = 44.05, *SD* = 11.57), and the authors reported an average of 1.9 children (*SD* = 0.79). The mean duration since diagnosis is 7.17 years (*SD* = 5.28). Of all participants, only 13 (6.5%) have reported previous suicide attempts (*M* = 1.77, *SD* = 3.44). We allowed participants to provide multiple responses for cancer types and treatment characteristics, leading to the total number of responses exceeding the number of surveyed individuals.

**Table 1:** Sociodemographic and cancer-related characteristics (*n* = 199)

Characteristic	*n*	Percentage
Gender		
Woman	199	100
Martial status		
Married	114	57.3
In a relationship, unmarried	39	19.6
No relationship	46	23.1
Children		
Yes	126	63.3
No	73	36.7
Professional status		
Employed	89	45.2
Student	49	24.9
On sick leave	6	3.0
Other (e.g., no activity/retirement)	53	26.9
Type of cancer		
Breast	121	60.8
Gynecological tumors	17	8.5
Intestine/rectum	6	3.0
Urological tumors (urinary tract, kidney, bladder, etc.)	4	2.0
Lung/bronchial tumors	4	2.0
Throat/nose/ears	5	2.5
Hematological diseases (leukemia, Hodgkin’s disease, non-Hodgkin’s lymphomas, etc.)	24	12
Skin (melanoma, basal cell carcinoma, etc.)	9	4.5
Soft tissue tumors (connective or fatty tissue, muscles, nerves, vessels, etc.)	3	1.5
Stomach, esophagus, pancreas	3	1.5
Central nervous system (brain, spinal cord, cranial nerves, glioblastoma, etc.)	7	3.5
Other tumor	15	7.5
Total	218	
Mean number of types of cancer	1.1	
Range	1–4	
Treatment in the last 2 months		
None	67	33.7
Surgery	26	13.1
Chemotherapy	48	24.1
Hormone therapy	49	24.6
Radiotherapy	18	9.0
Immunotherapy	17	8.5
Other treatment	43	21.6
Total	268	
Total number of treatments	201	
Mean number of treatments (all participants, *n* = 199)	1.01	
Mean number of treatments (participants in treatment, *n* = 132)	1.52	
Range of treatments	0–5	

Between August 2020 and November 2021, participants were recruited through social media and via invitations sent to psychotherapist networks, cancer support and awareness groups, and cancer treatment centers in North Rhine-Westphalia, Germany. Data collection was conducted through an anonymous online survey using SoSci-Survey (http://www.soscisurvey.de; [42]

Prospective participants were presented with an online consent form, which they had to acknowledge to confirm their informed consent. The study’s inclusion criteria were proficiency in the German language, a current or past diagnosis of cancer, and a minimum age of 18. The completion of the survey took roughly 60 min. To ensure participant safety, a 24/7 emergency hotline managed by the study team was provided, along with additional nationwide crisis intervention resources and hotlines. These resources were made available to respondents before, during, and after the survey. This study adhered to the Declaration of Helsinki and received approval from the local ethics committee (JCHF0067). It was registered with the German Clinical Trials Register (DRKS00020477; registered on June 30, 2020).^42^

### Measures

PB and TB were assessed using the Interpersonal Needs Questionnaire (INQ; German version [30]). The INQ encompasses two elements of the IPTS—“Thwarted Belongingness” (INQ-TB,consisting of nine items) and “Perceived Burdensomeness” (INQ-PB; comprising six items). All questions offer a 7-point Likert-type scale from 1 (“not relevant to me at all”) to 7 (“very applicable to me”). Validation studies conducted in Germany [30, 33] using student and population-representative samples revealed high internal consistencies for the scales (TB, *α* = 0.83 and 0.89; PB, *α* = 0.88 and 0.94). Internal consistencies in this study are *α* = 0.89, *ω* = 0.92 for the TB scale, and *α* = 0.88, *ω* = 0.92 for the PB scale.

Capability for suicide was assessed using the German Capability for Suicide Questionnaire (GCSQ; German version: [78]). The GCSQ is a self-report questionnaire that includes two scales: “Fearlessness about Death” and “Pain Tolerance,” comprised of ten items. In addition, the questionnaire contains an item that evaluates the individual’s self-perceived capability for suicide (“I could kill myself if I wanted to”),however, this item was not utilized in this study. Responses to the statements are recorded on a 5-point Likert-type scale (1 = “strongly agree” to 5 = “strongly disagree”). Psychometric studies of the GCSQ demonstrate medium to high internal consistency (*α* = 0.73–0.90; *ω* = 0.70–0.88) and high criterion validity for the pain tolerance subscale, as reported by Wachtel et al. [78] and Cwik et al. [16]. The present study demonstrated internal consistencies of *α* = 0.88 and *ω* = 0.90 (fearlessness about death) and *α* = 0.79 and *ω* = 0.82 (pain tolerance). Pain tolerance is exclusively associated with the fourth hypothesis, so using scores for additional analysis is not a component of this study.

SI and related behaviors were assessed using the Suicide Ideation and Behavior Scale (SSEV; [73]), which measures the frequency and intensity of SI and related behaviors over 4 weeks through 11 items (for example, “During the past four weeks, I wished to be dead”). These items are measured on a six-point Likert-type scale, ranging from 1 (“never”) to 6 (“every day, multiple times a day”). Higher scores signify more frequent or severe SI and behaviors. The questionnaire includes items addressing both passive and active SI and intentions, as well as behaviors related to suicide, such as talking about it or planning it. Additional items inquire about recent and lifetime suicide attempts, both of which use a binary (yes/no) response format. The number of past suicide attempts is also noted. The German version of the SSEV demonstrates strong validity and internal consistency (*α* = 0.77–0.92), attesting to its reliability as an assessment instrument. The internal consistencies in this study are *α* = 0.89 and *ω* = 0.93. A draft version of the questionnaire was used in this study. The manual and questionnaire for the verified version of the SSEV can be found at www.psychometrikon.de. The SSEV items used in this study are presented in a table format in the appendix (Appendix B).

Hopelessness was assessed using the entrapment subscale of the German version of the Short Defeat and Entrapment Scale (SDES, [36]) as a surrogate measure for feelings of hopelessness related to TB and PB. Entrapment is defined as a strong wish to escape an unbearable situation, accompanied by the belief that all possible means of escape are blocked [29, 36]. The SDES covers eight items, four of which evaluate feelings of defeat, such as “I feel defeated by life.” The remaining four items assess feelings of entrapment (“I would like to get away from who I am and start again”). Each item is scored on a Likert-type scale ranging from 1 (“not at all like me”) to 4 (“extremely like me”). The original English version of the SDES and the German translation have shown high internal consistency (*α* = 0.88–0.94 [31],and *α* = 0.83–0.88, respectively, depending on the sample and scale). Internal consistencies in this study are *α* = 0.77, *ω* = 0.81 for the defeat subscale, and *α* = 0.85, *ω* = 0.86 for the entrapment subscale. Numerous studies have shown a significant correlation between feelings of entrapment and hopelessness, suggesting that individuals experiencing high levels of entrapment are also likely to feel hopeless [59, 61, 72].

### Statistical analyses

All statistical analyses were conducted using R Version 4.2.1. The reported *p*-values pertain to two-tailed tests (multiple linear regression, Spearman’s rank correlation) and to ANOVA model comparisons (standard *F*-test), with statistical significance set at *p* < 0.05 for all analyses. Table 2 presents the descriptive statistics. The appendix tabulates the correlations among the study variables (Appendix C). To ensure the robustness of our analyses, we scrutinized several assumptions for our regression models. Multicollinearity was assessed using the tolerance and variance inflation factor values, and they proved to be acceptable (i.e., > 0.10 or < 10, respectively). The normality of residuals was visually inspected using Q-Q plots, and deviations from normality were noted but deemed acceptable, given the rare and skewed nature of suicidality data [64]. Linearity and homoscedasticity were evaluated through residual vs. fitted plots, which indicated some deviations but were not severe enough to warrant transformations. Influential points were identified using Cook’s distance and leverage plots, and sensitivity analyses confirmed that these points did not substantially distort the results. The subsequent section provides further details of the statistical analyses.

**Table 2:** Descriptive statistics of variables relevant to the analyses

Variable	Questionnaire	*n*	Mean	Standard deviation	Minimum	Maximum
Fearlessness about death	GCSQ-FAD	196	11.44	5.49	5	25
Perceived burdensomeness	INQ-PB	197	10.2	6.21	6	36
Thwarted belongingness	INQ-TB	197	25.86	12.3	9	63
Suicidal ideation and behavior	SSEV	194	12.21	3.19	11	34
Passive suicidal ideation	SSEV-Pass	194	2.63	1.49	2	12
Active suicidal ideation	SSEV-Act	194	2.23	0.8	2	10
Suicidal intent	SSEV-Int	194	2.1	0.49	2	6
Entrapment	SDES-E	193	8.44	3.66	4	18

*n* is smaller compared to the baseline sample due to missing data, *GCSQ-FAD*, German Capability for Suicide Questionnaire—Fearlessness about Death; *INQ-PB*, Interpersonal Needs Questionnaire—Perceived Burdensomeness; *INQ-TB*, Interpersonal Needs Questionnaire—Thwarted Belongingness; *SSEV*, Suicide Ideation and Behavior Scale; *SSEV-Pass*, Suicide Ideation and Behavior Scale—passive suicidal ideation; *SSEV-Act*, Suicide Ideation and Behavior Scale—active suicidal ideation; *SSEV-Int*, Suicide Ideation and Behavior Scale—suicidal intent; *SDES-E*, Short Defeat and Entrapment Scale – Entrapment

## Results

### Hypothesis 1

“Thwarted belongingness and perceived burdensomeness are proximal and sufficient causes of passive suicidal ideation.”

Passive SI, determined by relevant items of the SSEV, served as the criterion, with PB and TB functioning as the primary predictors. As depicted in Table 3, both PB and TB significantly predicted passive SI.

**Table 3:** Multiple linear regression equation predicting passive suicidal ideation (*n* = 194)

Predictors entered in set	*F*	*df*	Adjusted *R*^2^	*b*	*t*	*p*	*r*	*sr*
1	38.88	2, 191	0.28			** < 0.001**		
Perceived burdensomeness				0.079	4.070	** < 0.001**	0.477	0.282
Thwarted belongingness				0.032	3.316	**0.001**	0.498	0.233
2	26.86	3, 190	0.29			** < 0.001**		
Perceived burdensomeness				0.053	2.138	**0.034**	0.477	0.241
Thwarted belongingness				0.033	3.422	** < 0.001**	0.498	0.153
Perceived burdensomeness × thwarted belongingness				0.001	1.535	0.126	0.415	0.111

In bold: *p* < 0.05, *n* is smaller compared to the baseline sample due to missing data, *r* zero-order correlation, *sr* part correlation

Although not directly relevant to hypothesis 1, we conducted an additional exploratory investigation into the correlation between passive SI and the IPTS in a second step. We did this by adding the interactive variable PB by TB to the model, following the methods used by previous studies [11]. The results of this analysis are presented in the lower portion of Table 3. The addition of the PB by TB interaction to the model did not significantly increase its effectiveness in accounting for variations in passive SI, resulting in *F*(1, 190) = 2.356, *p* = 0.127.

### Hypothesis 2

“The simultaneous presence of thwarted belongingness and perceived burdensomeness, when perceived as stable and unchanging (i.e., hopelessness regarding these states), is a proximal and sufficient cause of active suicidal desire.”

We assessed predictors of active SI using pertinent items from the SSEV. A two-step multiple linear regression model, with active SI as the dependent variable, was constructed. The first procedural step incorporated TB and PB, measured by the INQ subscales, into the equation. At this juncture, we also input the score from the SDES Entrapment subscale, as the IPTS postulates that hopelessness regarding TB and PB is a prerequisite for active SI. As depicted in Table 4, this model significantly predicted active SI. We subsequently incorporated the two- and three-way interactions into the model (PB by TB; PB by hopelessness; TB by hopelessness; PB by TB by hopelessness). In this adjusted model, all interactions predicted active SI. This enhancement, adding the interactions to the model, significantly improved its effectiveness in accounting for variance in active SI, *F*(4, 185) = 17.266, *p* < 0.001. The explained variance increased by 19 percentage points.

**Table 4:** Multiple linear regression equation predicting active suicidal ideation (*n* = 193)

Predictors entered in set	*F*	*df*	Adjusted *R*^2^	*b*	*t*	*p*	*r*	*sr*
1	15.68	3, 189	0.20			** < 0.001**		
Perceived burdensomeness				0.025	2.152	**0.032**	0.385	0.154
Thwarted belongingness				0.006	0.939	0.309	0.380	0.068
Hopelessness				0.022	2.245	**0.026**	0.405	0.161
2	18.90	7, 185	0.39			** < 0.001**		
Perceived burdensomeness				− 0.096	− 1.547	0.124	0.385	0.005
Thwarted belongingness				0.000	0.066	0.947	0.380	0.008
Hopelessness				0.009	00.409	0.683	0.405	0.030
Perceived burdensomeness × thwarted belongingness				− 0.002	− 2.160	**0.032**	0.466	− 0.157
Perceived burdensomeness × hopelessness				− 0.008	− 2.202	**0.028**	0.471	− 0.160
Thwarted belongingness × hopelessness				0.006	3.954	** < 0.001**	0.527	0.280
Perceived burdensomeness × thwarted belongingness × hopelessness				0.000	5.061	** < 0.001**	0.578	0.349

In bold, *p* < 0.05, *n* is smaller compared to the baseline sample due to missing data*, r* zero-order correlation, *sr* part correlation

### Hypothesis 3

“The simultaneous presence of suicidal desire and lowered fear of death serves as the condition under which suicidal desire will transform into suicidal intent.”

A linear regression model was constructed, with suicidal intent, as gauged by relevant items from the SSEV, set as the criterion variable. Active SI and fearlessness about death were the primary predictors. Table 5 shows that active SI was a significant predictor of suicidal intent, while fearlessness about death was not.

**Table 5:** Multiple linear regression equation predicting suicidal intent (*n* = 194)

Predictors entered in set	*F*	*df*	Adjusted *R*^2^	*b*	*t*	*p*	*r*	*sr*
1	41.35	2, 191	0.29			** < 0.001**		
Active ideation				0.000	9.045	** < 0.001**	0.549	0.547
Fearlessness about death				0.000	− 0.333	0.739	0.057	− 0.024
2	39.19	3, 190	0.37			** < 0.001**		
Active ideation				0.436	10.786	** < 0.001**	0.549	0.616
Fearlessness about death				− 0.001	− 0.226	0.821	0.057	− 0.016
Active ideation × fearlessness about death				− 0.035	− 4.962	** < 0.001**	0.032	− 0.339

In bold, *p* < 0.05, *n* is smaller compared to the baseline sample due to missing data, *r* zero-order correlation, *sr* part correlation

We expanded the model in a second step by introducing the two-way interaction between active SI and fearlessness about death. This enhanced model showed a statistically significant interaction effect. With the inclusion of this interaction, the model’s effectiveness in accounting for variance in suicidal intent was significantly improved, *F*(1, 190) = 24.62, *p* < 0.001. The explained variance increased by 8 percentage points.

**Fig. 2:**
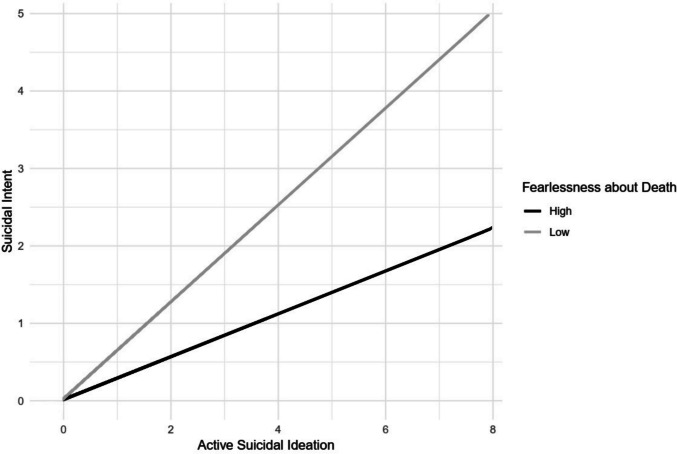
Interaction effect of active suicidal ideation and fearlessness about death on suicidal intent

To aid understanding, we developed an interaction diagram depicting the relationship between active SI and Fearlessness about death. Participants were grouped into “High” and “Low” fearlessness about death categories, determined by the 75th percentile cutoff for the “High” group and the 25th percentile for the “Low” group (Fig. 2).

## Discussion

The current study aimed to test the initial three hypotheses of the IPTS proposed by Van Orden et al. [76] within a cohort of female cancer patients. Firstly, the findings offer some support for the theory. Both PB and TB had positive correlations with passive SI, thereby confirming the first hypothesis. This finding aligns with discoveries from meta-analyses of the IPTS in other related samples [13, 46]. Furthermore, TB and PB were associated with active SI, particularly when interacting with hopelessness, consistent with evaluations of the IPTS in other populations [5, 6, 23]. Secondly, the findings corroborate the three-way interaction of TB, PB, and hopelessness as predictors of active SI. This finding aligns with previous studies that have identified a small yet significant interaction effect between unmet interpersonal needs and hopelessness on SI [32, 48, 74,80].

Regarding the third hypothesis, the IPTS suggests that the transition from SI to suicidal intent necessitates the simultaneous presence of suicidal desire and a reduction in the fear of death. Active SI was identified as a significant predictor of suicidal intent. A significant interaction between active SI and fearlessness about death was also discovered. Contrary to the hypothesis proposed by Van Orden et al. [76], our findings suggest that lower fearlessness about death (i.e., higher fear of death) is associated with higher suicidal intent. Cancer patients report various reasons for their death wish. Preserving self-determination in life’s final moments and maintaining or regaining control is a prominent reason [60]. Suicide (or assisted suicide) can be viewed as an act of retaining control in the final period of life. It has also been documented that female cancer patients, specifically those diagnosed with breast cancer, exhibit heightened levels of fear of death compared to the general cancer patient population [68]. These observations challenge the hypothesis proposed by the IPTS concerning this particular subgroup, as cancer patients tend to experience heightened fear of death rather than fearlessness, and may regard suicide as an act to regain or maintain control. While these findings provide valuable insights, several limitations must be considered when interpreting the results.

### Limitations and future directions

Notably, only a small number of participants reported suicidal desire (*n* = 24), and even fewer reported suicidal intent (*n* = 11). This limitation reduced the statistical power and obstructed a meaningful interpretation of results related to this hypothesis. While logistic regression or dichotomizing suicidal intent might seem appropriate for small samples, we opted for linear regression to retain the continuous nature of suicidal intent, as dichotomizing continuous variables can lead to loss of information and reduced statistical power [2]. However, bootstrapping methods were not applied due to the very small number of cases in the suicidal intent subgroup, which would limit the reliability of resampled estimates [18, 54]. A subgroup analysis including only participants with suicidal desires was deemed methodologically inappropriate due to the small sample size, which could potentially lead to unstable estimates and an increased risk of type I and II errors. Therefore, conclusions about this outcome should be interpreted with caution and considered exploratory. Future research with larger samples is needed to validate these findings and further explore the relationship between suicidal intent and the constructs of the IPTS.

Using regression analyses of cross-sectional data to evaluate hypotheses derived causally from the IPTS implies that our findings ought to be interpreted cautiously and validated in longitudinal research. This is even more relevant given that the IPTS proposes predicting future suicide risk [13], which is not feasible with our cross-sectional design.

Notably, we conducted our study’s recruitment phase during the early stages of the global pandemic incited by the SARS-CoV-2 virus, also known as coronavirus disease 2019 (COVID-19). Considering the observed increase in depression, anxiety, distress, and insomnia prevalence during the pandemic’s early phases and the heightened depression and anxiety risks among patients with chronic diseases, it is reasonable to suggest that the pandemic’s general effects could have influenced our study results [82]. Living alone appears to have lowered the quality of life for cancer patients during the pandemic [14], and on the whole, the pandemic resulted in a decreased sense of belonging, which could have also influenced the results concerning TB [49].

Another limitation pertains to the measurement tools used. The tools used to explore the essential principles of the IPTS (TB, PB, fearlessness about death) were all pre-established questionnaires designed initially to evaluate the theory’s primary elements. The measure used to assess hopelessness was not designed explicitly to gauge hopelessness regarding TB and PB, but rather, entrapment. This may limit the precision of our findings regarding the interaction of hopelessness with TB and PB. Regarding SI and suicidal intent, a preliminary version of the SSEV was put into use [73]. The initial evaluation of the questionnaire disclosed a two-factor structure composed of “cognitive engagement with suicide” and “action-related and externalizing experiences and behaviors relating to suicide.” However, the final version of the SSEV will contain four fewer items and display a unidimensional structure. It could be that this measurement does not sufficiently differentiate between active and passive SI and suicidal intent. For the evaluation of these variables, it would be beneficial for future research to consider using structured interviews, such as the revised Self-Injurious Thoughts and Behaviors Interview [24].

As stated in other research on suicide, suicide itself exemplifies a relatively low base rate event [64]. Our sample aligns with this observation, highlighting the need for larger samples in longitudinal validations of the theory to substantiate the hypothesis concerning suicidal behavior.

Finally, regarding the distribution of cancer diagnoses, the sample can only be considered partially representative of women in the 18–77 age range. The most common cancers for this age group are breast cancer, colorectal cancer, lung cancer, female reproductive tract cancers, and melanoma [22]. However, in our sample, breast cancer was the most prevalent, with gynecological tumors being the third most common. Furthermore, our sample differs from the general distribution of women in this age group, as only four participants (2%) reported lung cancer, contrasting with a higher number of participants reporting hematological diseases (*n* = 24, 12%). Moreover, those reporting breast cancer (*n* = 121, 60.8%) more than doubled the rate reported in German cancer statistics [22]. The uneven distribution of cancer diagnoses may be partially attributed to the age distribution of the sample population. In the age group corresponding to the sample’s mean age (*M* = 44.05 years, SD = 11.57), breast cancer is more prevalent, while lung cancer does not rank among the six most common types. Lung cancer is typically more prevalent in older patients, while breast cancer is the most common cancer in women of reproductive age [15, 25]. Generally, the characteristics of our sample align more closely with overall cancer statistics for childbearing-age females in Europe [22]. Nonetheless, it is noteworthy that our sample still has a higher proportion of participants diagnosed with breast cancer compared to the average (40.7 vs. 60.8%) [22]. The overrepresentation of breast cancer patients and the comparatively young age of our sample may limit the generalizability of our findings to female cancer patients with other tumor types or older age groups. Future research should include a more balanced distribution of cancer diagnoses and a broader age range to better reflect real-life demographics and validate these findings across diverse populations.

## Conclusion

As the first peer-reviewed test of the IPTS in a cancer cohort, this study provided valuable insights into the complex interplay between TB, PB, and hopelessness in predicting SI and behavior. When evaluated collectively, our findings provide partial support for the predictions suggested by the IPTS. The data indicated that both TB and PB were predictive factors of passive SI, while the combination of TB, PB, and hopelessness was predictive of active SI. We identified an interaction between active SI and fearlessness about death. Unexpectedly, a higher fear of death was associated with increased suicidal intent, contradicting theoretical assumptions. Given the efficacy of psychotherapy targeting fear of death in cancer patients [57], our findings highlight the potential clinical utility of integrating such approaches to address suicidality in this population. Assessing TB, PB, hopelessness, and fear of death equips therapists with a comprehensive framework to identify key risk factors for SI and suicidal crises, enabling tailored interventions for individual patients. While future research with larger samples is needed to validate these findings and further explore the relationship between suicidal intent and IPTS constructs in patients with cancer, the IPTS remains a valuable tool for health professionals in oncology. Given the limited literature connecting the IPTS and cancer [65], these findings represent a significant contribution to the current body of knowledge on this subject.

## Supplementary Information

Below is the link to the electronic supplementary material.

ESM 1 (DOCX 18.8 KB)

## Data Availability

The datasets used and analyzed during the current study are freely available at https://osf.io/935we/.
