# Supplementary material for: Testing the predictions of the interpersonal-psychological theory of suicide in a sample of female cancer patients
Source: Support Care Cancer. 2025 Dec 2;33(12):1157. doi: 10.1007/s00520-025-10224-2 (PMC12672701; doi:10.1007/s00520-025-10224-2)
Supplement: Supplementary file 1 — (DOCX 18.8 KB) [file 520_2025_10224_MOESM1_ESM.docx]

Appendix A

Fearlessness about Death = FAD

Interpersonal Needs Questionnaire = INQ

Interpersonal-Psychological Theory of Suicide = IPTS

Perceived Burdensomeness = PB

Short Defeat and Entrapment Scale = SDES

SDES-D = defeat subscale

SDES-E = entrapment subscale

SSEV = Suicide Ideation and Behavior Scale

SSEV-Act = active suicidal ideation

SSEV-Int = suicidal intent

SSEV-Pass = passive suicidal ideation

Suicidal Ideation = SI

The German Capability for Suicide Questionnaire = GCSQ

Thwarted Belongingness = TB

World Health Organization = WHO

Appendix B

*SSEV items used in this study*

| Item | Variable |
| --- | --- |
| During the past 4 weeks... |  |
| ...I thought it would be better if I wasn't alive. | SSEV-Pass |
| ...I wished I was dead. | SSEV-Pass |
| ...I thought about killing myself. | SSEV-Act |
| ...I have seriously considered killing myself | SSEV-Act |
| ...I had the intention of killing myself | SSEV-Int |
| ...I had the impulse to kill myself | SSEV-Int |

*Note.* SSEV-Pass = Suicide Ideation and Behavior Scale - passive suicidal ideation, SSEV-Act = Suicide Ideation and Behavior Scale - active suicidal ideation, SSEV-Int = Suicide Ideation and Behavior Scale - suicidal intent

Appendix C

*Correlations between study variables* (*n* = 193, case-wise deletion of missing data)

|  | GCSQ-PT | INQ-PB | INQ-TB | SSEV | SSEV-Pass | SSEV-Act | SSEV-Int | SDES | SDES-D | SDES-E |
| --- | --- | --- | --- | --- | --- | --- | --- | --- | --- | --- |
| GCSQ-FAD | **.142** | -.024 | -.051 | **.152** | **.169** | **.162** | .073 | **-.282** | **-.211** | **-.323** |
| GCSQ-PT | 1 | -.097 | **-.228** | -.065 | -.062 | -.065 | -.035 | **-293** | **-.286** | **-.265** |
| INQ-PB |  | 1 | **.579** | **.442** | **.447** | **.274** | .105 | **.575** | **.606** | **.494** |
| INQ-TB |  |  | 1 | **.404** | **.407** | **.340** | **.198** | **.723** | **.700** | **.683** |
| SSEV |  |  |  | 1 | **.938** | **.730** | **.489** | **.492** | **.514** | **.431** |
| SSEV-Pass |  |  |  |  | 1 | **.671** | **.431** | **.478** | **.499** | **.423** |
| SSEV-Act |  |  |  |  |  | 1 | **.661** | **.355** | **.388** | **.296** |
| SSEV-Int |  |  |  |  |  |  | 1 | **.196** | **.224** | **.159** |
| SDES |  |  |  |  |  |  |  | 1 | **.956** | **.952** |
| SDES-D |  |  |  |  |  |  |  |  | 1 | **.824** |
| SDES-E |  |  |  |  |  |  |  |  |  | 1 |

*Note.* Spearman rank correlations, In bold: *p*-values < .05*,* GCSQ-FAD = German Capability for Suicide Questionnaire - Fearlessness about Death, GCSQ-PT = German Capability for Suicide Questionnaire – Pain Tolerance, INQ-PB = Interpersonal Needs Questionnaire - Perceived Burdensomeness, INQ-TB = Interpersonal Needs Questionnaire - Thwarted Belongingness, SSEV = Suicide Ideation and Behavior Scale, SSEV-Pass = Suicide Ideation and Behavior Scale - passive suicidal ideation, SSEV-Act = Suicide Ideation and Behavior Scale - active suicidal ideation, SSEV-Int = Suicide Ideation and Behavior Scale - suicidal intent, SDES = Short Defeat and Entrapment Scale, SDES-D = Short Defeat and Entrapment Scale – Defeat, SDES-E = Short Defeat and Entrapment Scale – Entrapment
